# The Impact of 2-Ketones Released from Solid Lipid Nanoparticles on Growth Modulation and Antioxidant System of *Lactuca sativa*

**DOI:** 10.3390/plants12173094

**Published:** 2023-08-29

**Authors:** Paola Fincheira, Javier Espinoza, Joelis Vera, Daniela Berrios, Javiera Nahuelcura, Antonieta Ruiz, Andrés Quiroz, Luis Bustamante, Pablo Cornejo, Gonzalo Tortella, María Cristina Diez, Adalberto Benavides-Mendoza, Olga Rubilar

**Affiliations:** 1 Centro de Excelencia en Investigación Biotecnológica Aplicada al Medio Ambiente (CIBAMA), Facultad de Ingeniería y Ciencias, Universidad de La Frontera, Av. Francisco Salazar 01145, P.O. Box 54-D, Temuco 4811230, Chile; javier.espinoza@ufrontera.cl (J.E.); j.vera12@ufromail.cl (J.V.); andres.quiroz@ufrontera.cl (A.Q.); gonzalo.tortella@ufrontera.cl (G.T.); cristina.diez@ufrontera.cl (M.C.D.); olga.rubilar@ufrontera.cl (O.R.); 2 Departamento de Ciencias Químicas y Recursos Naturales, Universidad de La Frontera, Av. Francisco Salazar 01145, P.O. Box 54-D, Temuco 4811230, Chile; d.berrios01@ufromail.cl (D.B.); jnahuelcurav@gmail.com (J.N.); maria.ruiz@ufrontera.cl (A.R.); 3 Departamento de Análisis Instrumental, Facultad de Farmacia, Universidad de Concepción, P.O. Box 160-C, Concepción 4030000, Chile; lbustamante@udec.cl; 4 Escuela de Agronomía, Facultad de Ciencias Agronómicas y de los Alimentos, Pontificia Universidad Católica de Valparaíso, Calle San Francisco s/n, La Palma, Quillota 2260000, Chile; pablo.cornejo@pucv.cl; 5 Departamento de Ingeniería Química, Universidad de La Frontera, Av. Francisco Salazar 01145, P.O. Box 54-D, Temuco 4811230, Chile; 6 Departamento de Horticultura, Universidad Autónoma Agraria Antonio Narro, Saltillo 25315, Mexico; abenmen@gmail.com

**Keywords:** volatile organic compounds, lipid nanoparticles, growth stimulation, antioxidant capacity, antioxidant enzymes

## Abstract

2-Ketones are signal molecules reported as plant growth stimulators, but their applications in vegetables have yet to be achieved. Solid lipid nanoparticles (SLNs) emerge as a relevant nanocarrier to develop formulations for the controlled release of 2-ketones. In this sense, seedlings of *Lactuca sativa* exposed to 125, 375, and 500 µL L^−1^ of encapsulated 2-nonanone and 2-tridecanone into SLNs were evaluated under controlled conditions. SLNs evidenced a spherical shape with a size of 230 nm. A controlled release of encapsulated doses of 2-nonanone and 2-tridecanone was observed, where a greater release was observed as the encapsulated dose of the compound increased. Root development was strongly stimulated mainly by 2-tridecanone and leaf area (25–32%) by 2-nonanone. Chlorophyll content increased by 15.8% with exposure to 500 µL L^−1^ of 2-nonanone, and carotenoid concentration was maintained with 2-nonanone. Antioxidant capacity decreased (13–62.7%) in *L. sativa* treated with 2-ketones, but the total phenol concentration strongly increased in seedlings exposed to some doses of 2-ketones. 2-Tridecanone strongly modulates the enzymatic activities associated with the scavenging of H_2_O_2_ at intra- and extracellular levels. In conclusion, 2-ketones released from SLNs modulated the growth and the antioxidant system of *L. sativa,* depending on the dose released.

## 1. Introduction

The constantly growing human population and the need to enhance production require improving agricultural production yield. Mineral fertilizers are widely applied to increase food production, reaching a global consumption of 201.84 million tons [1]. Nevertheless, the massive and excessive application of mineral fertilizers has significantly deteriorated the soil quality, generating an adverse impact on the ecosystem and human health [2]. The harmful effects of the ecosystem include areal runoffs, metal accumulation, excessive soil N and reduction of soil organic matter, and soil erosion, leading to soil imbalance [3]. Therefore, the search for innovative alternatives has focused on developing environmentally sustainable products in agriculture, i.e., organic amendments, plant breeding programs, and microbial inoculants [4,5]. Unfortunately, they have not fully reached their objective and adequate effects to mitigate the current problems in agriculture. In this context, microbial volatile organic compounds (VOCs) have emerged as potential tools to mitigate the problems in modern agriculture [6,7].

Microbial VOCs are signal molecules with a lipophilic nature characterized by low molecular weight (<300 g mol^−1^) and low boiling point at high vapor pressure (0.01 kPa at 20 °C) [7,8]. The chemical structures of microbial VOCs can belong to alcohols, sulfides, alkanes, alkenes, ketones, and terpenes, among others [9]. It has been reported that VOCs modulate root and leaf characteristics, fruit production, plant morphological changes, and seed germination [10].VOCs are effective in inducing plant growth at the root and foliar level without direct contact through the regulation of physiological and metabolic pathways [9]. The effects of VOCs on the regulation of phytohormone pathways, nutrient balance, photosynthetic parameters, and metabolic activity have been proven in *Arabidopsis thaliana*, which has been widely used as a model plant. These effects are regulated by genetic expression associated with biological processes, molecular function, and cell components. Interestingly, microbial VOC studies have been extended to *Mentha piperita*, *Medicago sativa*, *Lactuca sativa*, *Medicago truncatula*, *Sorghum bicolor*, *Oryza sativa*, *Solanum lycopersicum*, *Zea mays*, and *Capsicum annum* [11]. Particularly, preliminary studies indicate that 2-nonanone and 2-tridecanone strongly induce growth in *L. sativa* by enhancing root development and foliar growth, but their hydrophobic natures prevent their direct application [12]. In addition, both 2-nonanone and 2-undecanone stimulated the increase in density of root hairs and their length on *L. sativa* to improve nutrition acquisition [13]. Hence, the formulation of a carrier to the controlled release of 2-nonanone and 2-tridecanone is necessary to prospect their application in vegetables, emerging the employment of nanotechnology.

The encapsulation of active compounds using nanotechnological tools is considered a smart technology to optimize their sustainable release and protection against environmental stresses [14,15]. Generally, nanomaterials refer to particles with at least one dimension from 1 to 100 nm, but nanoparticles (NPs) used as nanocarriers can reach 1000 nm in some of their dimensions. NPs as a controlled release system are characterized by their viability, stability, solubility, low toxicity, and encapsulation efficiency [16,17]. Particularly, solid lipid nanoparticles (SLNs) (Size: 50 to 1000 nm) contain a lipid core (0.1–30% and solid at room temperature) and surfactant (0.5–5%), which is adequate for the inclusion of hydrophobic compounds [18]. SLNs are systems that allow the sustainable release of hydrophobic compounds and the reduction of compound losses associated with the leaching and degradation produced by environmental factors [19,20]. Recently, SLNs have been widely applied in food science and technology due to their remarkable stability, showing their efficacy for developing new agricultural products due to competitive cost and easy manufacture [20]. Nevertheless, according to our knowledge, there is no background on the physiological impact of the antioxidant system of 2-nonanone and 2-tridecanone released from SLNs in *L. sativa* seedlings. Thus, this research was focused on (1) characterizing the SLNs used for the encapsulation of 2-nonanone and 2-tridecanone, (2) evaluating the release of 2-nonanone and 2-tridecanone from SLNs, and (3) determining the growth promotion and antioxidant system of seedlings of *L. sativa* treated with 2-nonanone and 2-tridecanone released from SLNs.

## 2. Results

### 2.1. SLN Characterization

**Figure 1:**
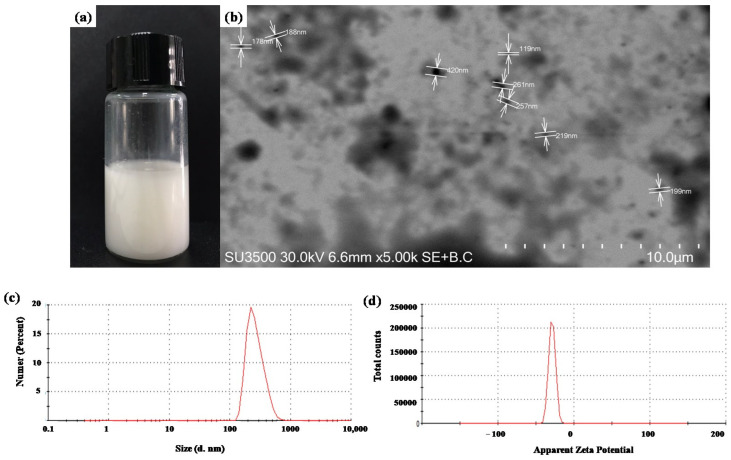
Microscopic characterization and dynamic light scattering analysis of solid lipid nanoparticles to encapsulate doses of 2-nonanone and 2-tridecanone. Representative photograph of SLNs dispersed (**a**) in water and (**b**) captured in a scanning transmission electron microscope and (**c**) hydrodynamic size and (**d**) stability measured by ζ-potential through dynamic light scattering.

SLNs were formulated to control the release of 2-nonanone and 2-tridecanone and their protection against environmental stresses. Figure 1a shows a photograph that evidences SLNs suspended in aqueous solution. The surface and physical morphology were analyzed by a scanning transmission electron microscope (STEM), where Figure 1b is a representative photograph that evidenced the spherical shape and homogeneous surface of SLNs homogeneously dispersed with a size range from 119 to 420 nm with a mean of 230 nm. The dynamic light scattering (DLS) technique shows a hydrodynamic mean size of 358.7 nm with a polydispersity index (PDI) of 0.26. Furthermore, the stability of SLN measurement through ζ-potential shows a value of −28.8 mV. Table 1 shows the properties of SLNs on months 1 and 2 after their formulation at 25 °C. A slight decrease in the size of particles for SLN formulation and those containing 2-nonanone and 2-tridecanone was observed. The encapsulation of compounds did not show an increase in the size of the formulation. The PDI and ζ -potential show that SLN formulation and SLN containing different doses of compounds remain homogeneous and stable for 2 months.

**Figure 2:**
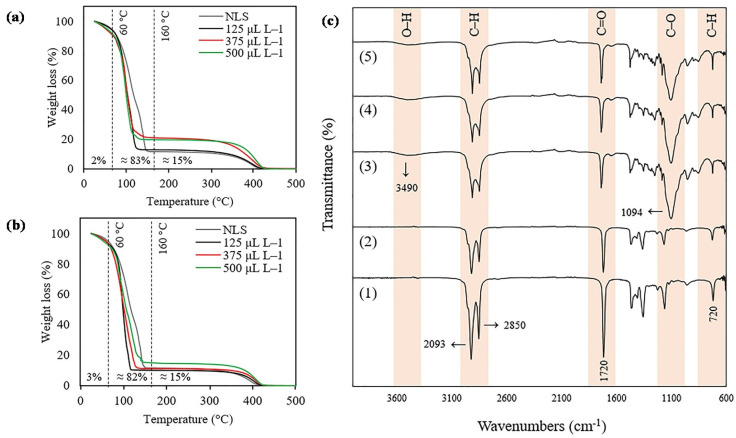
Physicochemical characterization of SLNs containing 2-ketones. TGA of SLNs containing (**a**) 2-nonanone and (**b**) 2-tridecanone. (**c**) FTIR spectra of (1) 2-tridecanone, (2) 2-nonanone, (3) SLNs containing 2-nonanone, (4) SLNs containing 2-tridecanone, and (5) formulation of SLN.

Thermogravimetric analysis (TGA) was performed to study the decomposition profiles and weight loss percentages of the SLNs caused by heating. The TGA results of all samples showed that the mass loss occurred in two steps. The first step occurred approximately between 60 and 160 °C, followed by the second step around 350 to 450 °C. The main weight loss (~83%) was observed for all the samples between 60 and 160 °C. High concentrations of 2-ketones tend to lose more of their weight (Figure 2a,b). Figure 2c shows the Fourier-transform infrared (FTIR) absorption spectra of 2-ketones and the SLN formulations containing 2-ketones. All spectra demonstrated a broad absorption peak located at approximately 2093 and 2850 cm^−1^ corresponding to single bond C-H stretching, which confirms the presence of the hydrocarbon chains of the 2-ketones and glyceryl tristearate. In addition, the peak located at 3490 cm^−1^ confirms the characteristic band of O-H vibration present in the structure of glyceryl tristearate. SLN formulation and 2-ketones showed an absorption peak at 1700 cm^−1^ corresponding to the stretching vibration double bond C=O. Additionally, the bands at 1094 belong to the stretching vibration of C-O present in the structure of glyceryl tristearate. Finally, the absorption peak at 720 cm^−1^ refers to C-H of 2-ketones and glyceryl tristearate.

### 2.2. Release of 2-Nonanone and 2-Tridecanone from SLN

**Figure 3:**
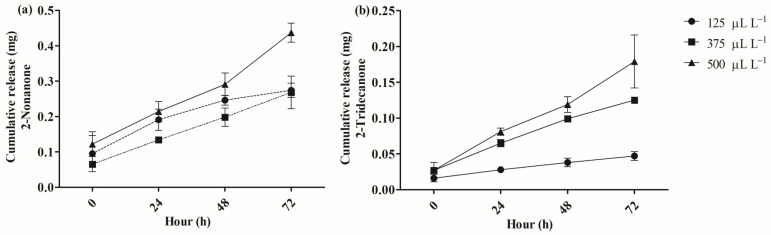
Cumulative release of (**a**) 2-nonanone and (**b**) 2-tridecanone from solid lipid nanoparticles (SLNs) during 72 h at 25 °C. The points represent a mean of three replicates, and bars represent standard error.

Figure 3 shows the controlled release of encapsulated doses (125, 375, or 500 µL L^−1^) of 2-nonanone and 2-tridecanone from SLNs during 72 h. A greater release was observed as the encapsulated dose of the compound increased. Encapsulated doses of 2-nonanone at 125, 375, and 500 µL L^−1^ released 0.13, 0.19, and 0.21 mg at 24 h and 0.26, 0.27, and 0.43 mg at 72 h (Figure 3a). Furthermore, encapsulated doses of 2-tridecanone at 125, 375, and 500 µL L^−1^ released 0.016, 0.026, and 0.027 mg at 24 h and 0.047, 0.12, and 0.17 mg at 72 h (Figure 3b). The results indicated a more significant release of 2-nonanone compared with 2-tridecanone (about three times).

### 2.3. Plant Growth

**Figure 4:**
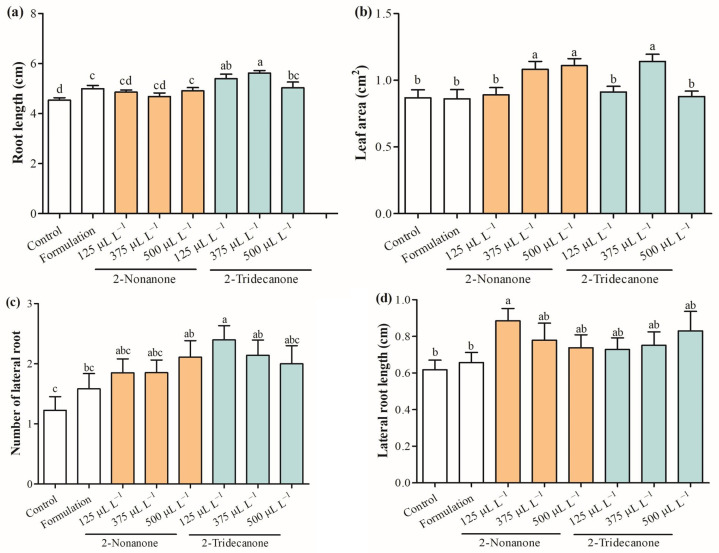
Growth promotion on *L. sativa* seedlings on day 14 modulated by different doses of 2-nonanone and 2-tridecanone released from SLNs. (**a**) Root length, (**b**) leaf area, (**c**) number of lateral root length, and (**d**) lateral root length. Letters indicate statistically significant differences according to the ANOVA test (*p* < 0.05, Tukey test) (N = 20–30). Bars indicate standard error.

Figure 4 shows the growth promotion at the root and foliar levels modulated by different doses of 2-nonanone and 2-tridecanone release from SLN. The results indicated that root length increased by 18.9% and 24.0% regarding control with the exposure to SLNs containing 125 and 375 µL L^−1^ of 2-tridecanone, and similar results were obtained by treatments with 2-nonanone considering control (Figure 4a). Moreover, the foliar area was stimulated in 25.5% and 27.9% by 2-nonanone (375 and 500 µL L^−1^) released from SLNs as well as in 32.5% with the exposure to 375 µL L^−1^ of 2-tridecanone encapsulated in SLNs (Figure 4b). Interestingly, lateral roots increased strongly (75.4–96.7%) with the exposure to SLNs containing 125 and 375 µL L^−1^ of 2-tridecanone (Figure 4c). The lateral root length was stimulated in 44.2% only with the exposure to encapsulated 2-nonanone (125 µL L^−1^) (Figure 4d).

### 2.4. Photosynthetic Pigments and Physiological Parameters

**Figure 5:**
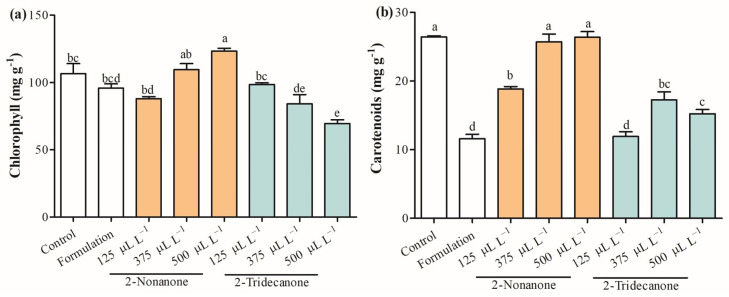
Total (**a**) chlorophyll and (**b**) carotenoid contents in leaves of *L. sativa* exposed to doses of 2-nonanone and 2-tridecanone released from SLNs on day 14. Letters indicate statistically significant differences according to the ANOVA test (N = 3, *p* < 0.05, Tukey test).

**Figure 6:**
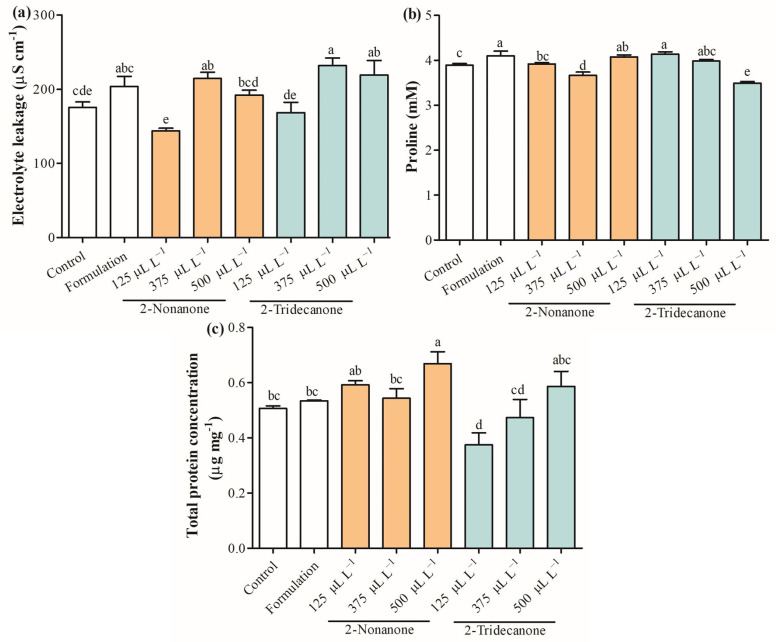
(**a**) Electrolyte, (**b**) leakage, (**c**) proline, and total protein concentration on seedling leaves of *L. sativa* exposed to doses of 2-nonanone and 2-tridecanone released from SLNs on day 14. Letters indicate statistically significant differences according to the ANOVA test (N = 3, *p* < 0.05, Tukey test).

Figure 5 shows physiological parameters evaluated on seedlings of *L. sativa* exposure to 2-nonanone and 2-tridecanone released from SLNs. The total chlorophyll content was enhanced in 23.4% only in seedlings treated with SLNs containing 500 µL L^−1^ of 2-nonanone (Figure 5a). Curiously, the total carotenoid content decreased by 28.8%, 55.0%, and 56.2% in seedlings exposed to SLN formulation and SLNs containing 2-nonanone and 2-tridecanone, respectively (Figure 5b). The electrolyte leakage was enhanced by 22.4%, 32.4%, and 25.0% in seedlings exposed to 2-nonanone (375 µL L^−1^) and 2-tridecanone (375 and 500 µL L^−1^) released from SLNs (Figure 6a). The proline content increased in *L. sativa* seedlings treated with the SLN formulation, and 2-nonanone (500 µL L^−1^) and 2-tridecanone (125 µL L^−1^) released from it by 5.3%, 6.1%, 4.6%, and 2.3%, and a decrease by 6% and 10.3% regarding control (Figure 6b). The total protein concentration was increased by 32% in seedlings exposed only to SLNs containing 500 µL L^−1^ and slightly increased in seedlings treated with encapsulated 125 µL L^−1^ and 500 µL L^−1^ of 2-nonanone (Figure 6c).

### 2.5. Antioxidant Capacity

**Figure 7:**
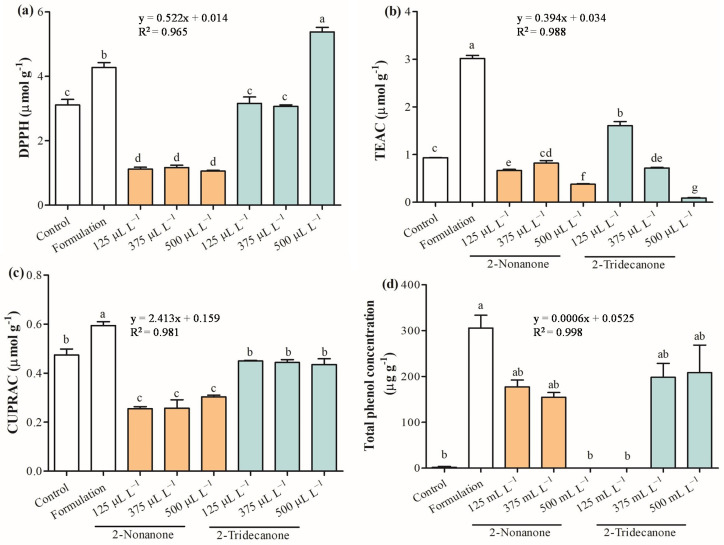
Antioxidant activity evaluated in leaves of *L. sativa* mediated by doses of 2-nonanone and 2-tridecanone released from SLNs on day 14. (**a**) 2,2-Diphenyl-1-picrylhydrazyl (DPPH) radical, (**b**) Trolox equivalent antioxidant activity (TEAC), (**c**) cupric ion-reducing antioxidant activity (CUPRAC), and (**d**) total phenol concentration. Letters indicate statistically significant differences according to the ANOVA test (N = 3, *p* < 0.05, Tukey test).

**Figure 8:**
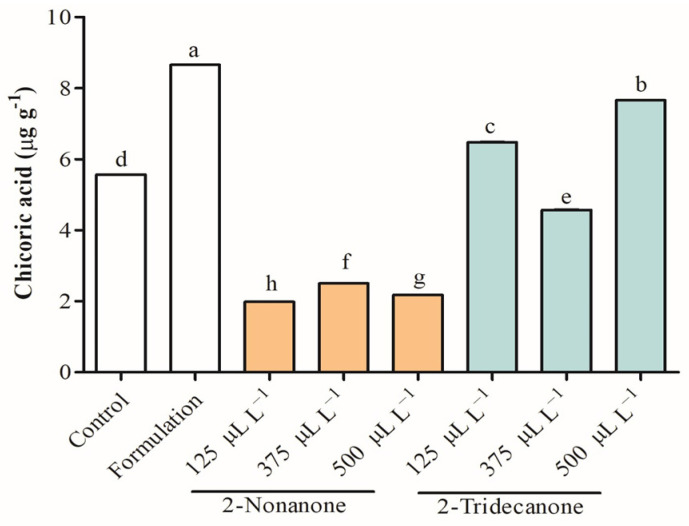
Chicoric acid identification and concentration in leaves of *L. sativa* seedlings exposed to doses of 2-nonanone and 2-tridecanone released from SLNs. Letters indicate statistically significant differences according to the ANOVA test (N = 3, *p* < 0.05, Tukey test).

Antioxidant capacity constitutes an essential parameter to evaluate the plant defense system. 2,2-Diphenyl-1-picrylhydrazyl(DPPH) radical, Trolox equivalent antioxidant activity (TEAC), and cupric ion-reducing antioxidant activity (CUPRAC) were assessed to provide a complete study about the elimination of free radicals in seedlings of *L. sativa* treated with 2-nonanone and 2-tridecanone released from SLNs (Figure 7). The antioxidant capacity decreased strongly in treatments of *L. sativa* exposed to doses of 2-nonanone, where DPPH was reduced to 62.7%, TEAC between 13.0% and 60.3%, and CUPRAC from 36.1% and 46.8% compared with control. Additionally, *L. sativa* seedlings exposed to 2-tridecanone release from SLNs tended to maintain antioxidant capacity or increase in specific cases. Remarkably, *L. sativa* seedlings exposed to encapsulated 125 and 500 µL L^−1^ 2-tridecanone increased DPPH and TEAC by 72.0%, respectively. Interestingly, as the dose of encapsulated 2-tridecanone increases, the antioxidant activity in the TEAC test decreases. Meanwhile, all doses of 2-tridecanone remained similar to the control in the CUPRAC test. Finally, the total phenol concentration was increased in seedlings exposed to SLN formulation and was strongly increased in determined treatments with 2-nonanone (125 and 375 µL L^−1^) and 2-tridecanone (375 and 500 µL L^−1^). Curiously, phenols were not detected in seedlings exposed to 500 and 125 µL L^−1^ of 2-nonanone and 2-tridecanone, respectively. The control presented 2 µg g^−1^ of total phenols, and its concentration was strongly increased in plants exposed to 2-nonanone (125 and 375 µL L^−1^) and 2-tridecanone (500 and 125 µL L^−1^) released from SLN. Chicoric acid belonging to hydroxycinnamic acids was detected and identified in *L. sativa* leaves based on their UV–VIS and tandem mass spectra, according to [M−H]^−^ (m/z) 473.1, a product ion (m/z) of 311.0, and λ_max_ of 329 nm. The chicoric acid concentration was increased in 55.5% in seedlings exposed to the SLN formulation and encapsulated 2-tridecanone between 16.3% and 37.7%. A substantial decrease in chicoric acid was observed in *L. sativa* exposed to 2-nonanone released from SLNs (~60%) (Figure 8).

### 2.6. Antioxidant Enzymes

**Figure 9:**
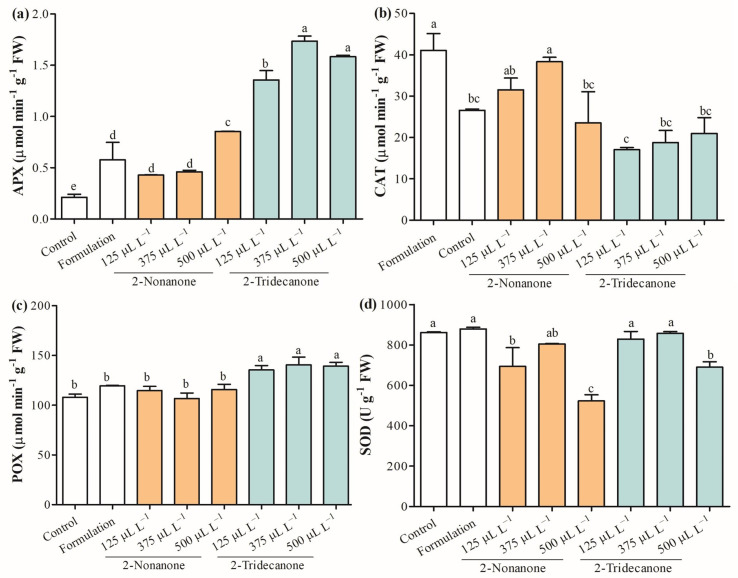
Antioxidant enzymes evaluated on seedlings of *L. sativa* mediated by 2-nonanone and 2-tridecanone released from SLNs on day 14. (**a**) Ascorbate peroxidase (APX), (**b**) catalase (CAT), (**c**) peroxidase (POX), and (**d**) superoxide dismutase (SOD). Letters indicate statistically significant differences according to the ANOVA test (N = 3, *p* < 0.05, Tukey test).

Reactive oxygen species (ROS) are naturally generated during cellular metabolism in plants, whereby the antioxidative enzymatic system is activated to counteract their effect. Figure 9a shows that *ascorbate peroxidase* (APX) (EC 1.11.1.11) increased between 100% and 304.7% in seedlings treated with 2-nonanone released from SLNs. Furthermore, seedlings exposed to encapsulating 2-tridecanone enhanced the APX range from 542.8% to 652.3%, as the concentration increased. *Catalase* (CAT) (EC 1.11.1.6) activity decreased in seedlings treated with 2-nonanone (500 µL L^−1^) by 11.4%, and those exposed to 2-tridecanone from 21.1% to 35.8%, depending on the concentration, with respect to control (Figure 9b). *Peroxidase* (POX) (EC 1.11.1.7) was stimulated in the range of 25.3% to 30.0% in *L. sativa* seedlings exposed to 2-tridecanone release from SLNs, and there were no significant changes with exposure to 2-nonanone (Figure 9c). Additionally, *superoxide dismutase* (SOD) (EC 1.15.1.1) decreased in seedlings exposed to 2-nonanone by 19.5% and 39.3% at the encapsulated doses of 125 and 500 µL L^−1^, respectively (Figure 9d).

## 3. Discussion

The harmful effects of mineral fertilizers on soil quality and human health have led to the search for new alternatives [21,22,23]. Thus, microbial VOCs have emerged as a potential tool to reduce the use of chemical fertilizers due to their essential effects on stimulating plant growth [9,23]. VOCs are considered secondary metabolites with ecological relevance due to their role in plant adaptability to global climate change and survival [24]. Studies performed in *A. thaliana* as a model plant has revealed that VOCs directly promote plant growth due to their effects on hormonal signaling, nutrition, and modulation of photosynthesis, among other metabolic processes. Previous studies have revealed that 2-nonanone and 2-tridecanone increase the root elongation and seed germination of *L. sativa* during the germination stage [25]. Moreover, these 2-ketones evidenced a stimulating effect on the root length, shoot length, dry weight, and root development of *L. sativa* [12]. The 2-ketones used in this study have shown an increase in the density of root hairs and their length on *L. sativa* to improve nutrition acquisition [12]. Therefore, the development of an effective carrier must be formulated for the controlled release of these 2-ketones applied in agricultural systems.

SLNs have several advantages compared with other polymeric nanoencapsulation techniques; i.e., they reduce losses through leaching, volatilization, and degradation [20]. This study formulated SLNs with glyceryl tristearate as a solid lipid due to its high biocompatibility and lower toxicity [26,27]. SLNs evidenced a spherical shape and regular surface with a mean size of 230 nm in concordance with a previous study by Ludtke et al. [28]. Moreover, the spherical shape and homogeneous surface of SLNs were confirmed by STEM analysis, which agrees with the study carried out by Oehlke et al. [29] and Fincheira et al. [30]. ζ-potential plays an important role in determining the stability of an SLN suspension, where high values should be achieved to form a high energy barrier, favoring good stability [31,32]. ζ-potential for SLNs (−28.8 mV) indicates good stability, and a PDI value of 0.26 evidences a remarkable particle homogeneity of SLNs, potentiating their application in the agricultural field. Furthermore, the hydrodynamic size, PDI, and ζ-potential evaluated during the 2 months revealed the excellent stability of SLNs to encapsulate all doses of 2-nonanone and 2-tridecanone, maintaining their properties under environmental conditions. In addition, TGA supported that SLN formulation and those containing the different doses of 2-nonanone and 2-tridecanone showed great thermal stability, where, at ~50 °C, there was only 3% mass loss. FTIR measurements supported the efficient encapsulation of 2-ketones into SLNs composed of glyceryl tristearate with characteristic functional groups. In this sense, the physicochemical characterization evidences that SLNs constitute an effective and stable nanocarrier to encapsulate 2-ketones with potential application in agriculture.

The headspace autosampler TurboMatrix 40 coupled to a Clarus 680 gas chromatograph with a flame ionization detector (HS-GC-FID-Headspace) was used to evaluate the capacity of SLNs to release different doses of 2-ketones over time [33]. The release of 2-nonanone and 2-undecanone increased as the encapsulated dose increased, remaining constant for 72 h. This suggests that both compounds interact similarly with the lipid matrix, independent of the encapsulated dose at 125 and 500 µL L^−1^. It noteworthy that doses of 2-tridecanone showed a lower release than 2-nonanone, which can be explained by the higher affinity and retention of the long carbon–ketone chain (13 carbons) with the lipid matrix [30,33]. Hence, the volatilization of 2-tridecanone is prevented by the highest number of carbon and molecular weights due to its greater stability inside the lipid matrix. These results support the great capacity of SLNs to release in a controlled manner 2-nonanone and 2-tridecanone under environmental conditions, in concordance with that reported by Fincheira et al. [33]. Therefore, SLNs exhibited interesting physicochemical properties and a remarkable capacity to release 2-ketones, allowing the prospect of their application in vegetables.

Previous studies showed relevant evidence that those doses from 125 to 500 µL L^−1^ of 2-nonanone and 2-tridecanone released from lipid NPs significantly increase the growth of *L. sativa* and *Lycopersicum esculentum* with exposure during 10 days [30,33]. Nevertheless, there are no known studies covering 14-day exposure and its physiological effects on the antioxidant system. The growth promotion activities of encapsulated doses (125, 375, and 500 µL L^−1^) of 2-nonanone and 2-tridecanone were tested in seedlings of *L. sativa* for 14 days. The life and adaptation of plants strongly depend on the strength of their root system [34,35]. The root system has an essential role in acquiring nutrients, water, and organic salt [34,35]. Therefore, a radical system is pivotal to plant soil exploration under climate change conditions [34]. The results strongly evidence that 2-ketones released from SLNs modulate root system growth, depending on the encapsulated dose, highlighting mainly that 2-tridecanone (125 and 375 µL L^−1^) has an important activity to stimulate the growth of the principal root and the number of lateral roots. This suggests that 2-ketones exert an action on root development at a specific dose, likely due to a possible action on the modulation of phytohormone pathways or physiological processes related to the growth of radical cells [36]. Additionally, the stimulation of root development may be associated with greater acquisition of nutrients by a more significant surface exploration [34,35]. A study performed by Zhang et al. [37] evidenced that root development is strongly associated with the upregulation of gene expression for auxin synthesis in the aerial region and the decrease of auxin concentration in leaves and its increase in the root region, favoring the basipetal auxin transport. Moreover, 2-nonanone (375 and 500 µL L^−1^) and 2-tridecanone (375 µL L^−1^) improved the leaf growth of *L. sativa* seedlings. The data indicated that 375 µL L^−1^ of 2-tridecanone encapsulated into SLNs was the most efficient dose to improve the growth of *L. sativa* seedlings in 15 days. It has previously been reported that the increase in leaf area triggered by volatiles is due to the regulation of cytokinin receptor and cytokinin and ethylene pathways in *A. thaliana*, which suggests a possible action through these phytohormones [38]. However, further studies must be carried out to reveal the mechanism of action activated by 2-ketones on root and foliar regions.

On the other hand, leaf chlorophyll content has a vital role in nutrient status, being an important indicator of plant health and having an important role as a central pigment for photosynthesis and plant growth [39]. In this study, chlorophyll content only was increased in seedlings of *L. sativa* exposed to 500 µL L^−1^ of 2-nonanone encapsulated into SLNs and decreased in plants exposed to high concentrations of 2-tridecanone, suggesting a possible stress induced by it. Furthermore, carotenoids are isoprenoid pigments produced by plants with an important role in protecting the photosynthetic system from ROS and light harvesting as auxiliary pigment [40]. Curiously, the concentration of carotenoids is maintained or strongly decreased at all encapsulated doses of 2-tridecanone, which can be influenced by the formulation of SLNs. 2-Ketones released by the SLNs probably interfere with the carotenoid biosynthesis pathway or produce plant stress, but more studies are needed. Therefore, a series of physiological parameters that reflect a response to stress by plants were studied.

Electrolyte leakage is a practical method to indirectly measure a plant cell’s stress response to biotic or abiotic stress and damage [41]. The results suggested that SLN formulation tends to increase electrolyte leakage in *L. sativa* seedlings relative to control. It could also be observed that the lowest encapsulated concentration of 2-ketone tends to decrease electrolyte leakage from plants. The treatments with more significant leakage of electrolytes coincide with those that tend to reduce the concentration of photosynthetic pigments, suggesting a possible stress response. Proline constitutes a plant defense mechanism against the harmful effects derived from adverse environmental conditions [42]. Proline is an amino acid relevant in the determination of protein structures and membranes and scavenges ROS [43]. According to our knowledge, no reports associate an effect of microbial volatiles with the concentration of proline in plants. The results obtained in proline concentration indicate that SLN formulation produces a stress response in *L. sativa*, being similar to seedlings treated with encapsulated 2-nonanone (500 µL L^−1^) and 2-tridecanone (125 and 375 µL L^−1^). Despite the increase in electrolyte leakage and the proline concentration in the SLN formulation and some treatments, the total protein concentration in seedlings is maintained and even increases in some treatments. Generally, protein quantification is widely used to reflect the nutrient status [44]. This indicates the capacity of *L. sativa* seedlings to respond to the possible stress triggered by SLN formulation and the release of 2-ketones without altering the main metabolic pathways. The following analyses were based on determining the effect of the SLN formulation and the release of the doses of 2-ketones on the antioxidant system of *L. sativa* seedlings.

In general, plant stresses alter a plant cell´s homeostasis and induce an osmotic disturbance, leading to the accumulation of ROS. The production and accumulation of ROS trigger damage in DNA, lipids, and proteins, affecting normal cellular functioning [45,46]. In this sense, the antioxidant system is the first line against the damage produced by ROS, such as O_2_^•–^, •OH, H_2_O_2_, and ^1^O_2_. Hence, studying antioxidant capacity is essential to evaluate the plant defense system to scavenge the elimination of ROS. DPPH radical scavenging is an accepted technique that allows detecting antioxidant activity due to its hydrogen donating ability [47]. TEAC is a simple assay widely applied in the study of water-soluble and lipid-soluble antioxidants through electron transfer [48]. The CUPRAC assay based on the Cu(II)-Cu(I) reduction in the presence of neocuproine is widely used to detect hydrophilic and lipophilic antioxidants through electron transference. The antioxidant capacity of leaves of *L. sativa* measured through DPPH, TEAC, and CUPRAC demonstrates that the SLN formulation increases the antioxidant capacity, and the release of the different doses of 2-nonanone from SLNs strongly decreases the antioxidant activity. This suggests that the plant stimulates its defenses in the presence of the SLN formulation. Interestingly, the release of 2-nonanone reduces the antioxidant capacity, probably due to an interaction with a cellular receptor of *L. sativa*. It should be noted that 2-nonanone is a natural VOC emitted by soil microorganism and reported as a plant growth regulator. Therefore, 2-nonanone constitutes a signal molecule that interacts with plant receptors to elicit a physiological response to promote the growth and development of *L. sativa* by decreasing its antioxidant capacity, exerting a specific response. This supports that 2-nonanone has an essential role in the interaction of seedlings of *L. sativa* with the environment and organisms that release this compound, playing an interesting ecological role. In addition, 2-nonanone can represent a molecule that allows an ecological adaptation to improve plant performance under environmental stress. Meanwhile, the release of doses of 2-tridecanone from SLNs tends to maintain antioxidant capacity according to the evaluation of DPPH and CUPRAC, where only the encapsulation of 500 µL L^−1^ of 2-nonanone increased DPPH. Conversely, TEAC evidenced that 125 µL L^−1^ of 2-tridecanone increases the antioxidant capacity, and as the encapsulated dose concentration increases, the antioxidant activity decreases. This result suggests that 2-nonanone and 2-tridecanone have a differential role in *L. sativa* response, determining a differential response pattern at the ecological community level. Otherwise, the Folin–Ciocalteu method is a methodology to determine total polyphenol concentration through a spectrophotometric technique, which has been linked with a high antioxidant capacity [49]. Phenolic compounds are characterized by low molecular weight and a single aromatic ring or more complex molecular, such as flavonoids. Hydroxybenzoic acids and hydroxycinnamic acids are the principal subgroups of phenolic acids associated with nutrient absorption and protein synthesis [50]. Interestingly, the releases of 2-nonanone (125 and 375 µL L^−1^) and 2-tridecanone (375 and 500 µL L^−1^) released from SLNs increase considerably the total phenol concentration, suggesting their capacity to prevent the oxidative process by scavenging free radicals and ROS [51]. It should be noted that the concentration of total phenols in the control and in those plants exposed to the release of 2-nonanone (500 µL L^−1^) and 2-tridecanone (125 µL L^−1^) from SLNs was not detected through this methodology. Particularly, chicoric acid (C_22_H_18_O_12_) belongs to caffeic acid derivatives with an important antioxidant activity and role in regulating lipid and glucose metabolism, which was identified in sample extracts [52]. Interestingly, the concentration of chicoric acid was increased in seedlings exposed to 2-tridecanone (125 and 375 µL L^−1^) released from SLNs and decreased strongly in treatments of seedlings exposed to 2-nonanone. This result suggests that phenolic compounds and specifically chicoric acid are highly dependent on the treatment applied to *L. sativa*. The presence of chicoric acid in seedlings exposed to treatments of 2-nonanone and 2-tridecanone at 500 and 125 µL L^−1^, respectively, suggests a greater sensitivity of the high performance liquid chromatography (HPLC) technique for the detection and quantification of phenolic compounds concerning the Folin spectrophotometric technique.

Additionally, we evaluated the enzymatic components of the antioxidant defense system due to their essential role in minimizing and scavenging the ROS concentration [46]. APX (1.11.1.11) utilizes ascorbate as a specific electron donor to scavenge H_2_O_2_ to H_2_O and dehydroascorbate in the chloroplast and cytosol, being an integral component of the ascorbate–glutathione (ASC–GSH) cycle [46]. Additionally, CAT (1.11.1.6) catalyzes the dismutation of H_2_O_2_ into H_2_O and O_2_ predominantly in peroxisomes during the processes of β-oxidation of fatty acids and photorespiration [53]. APX is strongly increased in *L. sativa* seedlings exposed to all treatments, even in those exposed only to the SLN formulation, highlighting mainly the seedlings exposed to the released doses of 2-tridecanone. This suggests a possible role of 2-tridecanone in inducing scavenging of H_2_O_2_, mainly in chloroplast and cytosol through APX. Contrarily, CAT strongly decreases its activity in seedlings exposed to 2-tridecanone released from SLNs, while 2-nonanone also tends to decrease its activity. This suggests less activity to scavenge H_2_O_2_ in peroxisomes. Moreover, POX (EC 1.11.1.7) acts in the extracellular space to scavenge H_2_O_2_ [46]; this enzyme activity was increased in seedlings exposed to 2-tridecanone, suggesting its role in actively scavenging H_2_O_2_ at the extracellular level. Finally, SOD (EC 1.15.1.1) dismutes O_2_^•–^ into H_2_O_2_, reducing the •OH formation [54]. The results indicated that SOD activity decreases in seedlings exposed to a high dose of 2-ketone, being maintained by the other treatments. In general, the results showed that mainly 2-tridecanone strongly modulate the enzymatic activities associated with the scavenging of H_2_O_2_ at the intra- and extracellular levels.

## 4. Materials and Methods

### 4.1. Formulation of SLN Containing 2-Nonanone and 2-Tridecanone

SLNs containing 125, 375, or 500 µL L^−1^ of 2-nonanone or 2-tridecanone were synthesized by high shear homogenization, followed by an ultrasonication method [30,55]. The lipid phase composed of glyceryl tristearate (Sigma-Aldrich^®^) (5% *w*/*v*) was dissolved in 4 mL of hexane (MerckMilipore^®^, Bedford, MA, USA) at 70 °C. Subsequently, 125, 375, or 500 µL L^−1^ of 2-nonanone or 2-tridecanone (Sigma-Aldrich, St. Louis, MO, USA) was added to the lipid phase. The aqueous phase of Tween 80 at 1.5% *w*/*v* was dissolved in 40 mL of distilled water at 70 °C. The aqueous phase was gradually added to the lipid phase and homogenized at 10,000 rpm for 5 min using Ultraturrax OV5 (Velp^®^, Scientifica, Usmate Velate, Italy). Then, the resulting solution was sonicated with an ultrasonic processor (Sonics and Materials, Newtown, CT, USA) for 5 min (5 s on/off, 35% amplitude) and cooled at 4 °C. Then, SLNs containing 2-ketones were kept at 4 °C until their use (vertical refrigerated cabinet, 2–8 °C, 290 L, HYC-290, Haier^®^, Qingdao, China).

### 4.2. Characterization of Solid Lipid Nanoparticles

The morphology and particle size of SLNs were determined by STEM using a HITACHI SU3500 scanning electron microscope (Hitachi^®^, Tokyo, Japan). Additionally, SLNs were characterized by their size, polydispersity index (PDI), and surface charge (ζ-potential) using dynamic light scattering (DLS) (Zetasizer Nano ZS90, Malvern Instruments, Inc., Malvern, UK). TGA was carried out using Simultaneous Thermal Analyzer (STA) 6000 (PerkinElmer, Waltham, MA, USA), where ~20 mg of sample was placed into a ceramic pan and then exposed to a nitrogen atmosphere from 25 to 500 °C at a heating rate of 15 °C min^−1^. FTIR was carried out using an Agilent Technologies Cary (Santa Clara, CA, USA) 630 FTIR spectrophotometer, where the spectra were obtained with the transmittance between the frequencies of 3600 and 600 cm^−1^ [33].

### 4.3. Quantification of 2-Nonanone and 2-Tridecanone Released from SLN by HS-GC-FID

The quantity of a bioactive compound released from SLNs containing 125, 375, and 500 µL L^−1^ of 2-nonanone and 2-tridecanone was measured using HS-GC-FID (PerkinElmer, Waltham, MA, USA). The release curves of 2-ketones encapsulated in SLNs were performed for 72 h in triplicate. Aliquots of 300 µL of formulations containing 2-ketones were measured each 24 h and diluted in 1 mL of hexane. Then, samples were added to a 22 mL vial and heated for 1 min at 80 °C, and 3 mL of the head-space passing through the transfer line (130 °C) was injected into GC-FID for the quantification. The analysis was performed using a BPX5 capillary column (30 m length, 0.25 μm film thickness, ×0.25 mm) (SGE Forte, Trajan Scientific, and Medical, Ringwood, VIC, Australia). The operating conditions were as follows: injector temperature, 250 °C; carrier gas, He at 2.00 mL min^−1^; and oven temperature program, 40 °C for 0 min, increasing to 250 °C at 15 °C min^−1^, followed by 250 °C for 2 min. Detector temperature, 250 °C; gas, H_2_ at 45.00 mL min^−1^ and air at 450.00 mL min^−1^. The quantification of 2-nonanone and 2-tridecanone was performed by external calibration curves with commercial standards purchased from Sigma-Aldrich^®^ (Steinheim, Germany) [30,33].

### 4.4. Plant Experiment and Growth Conditions

Seeds of *L. sativa* (green lettuce cv Reina de Mayo Asepo) were used to evaluate the growth modulation of 2-nonanone and 2-tridecanone released from SLNs. Previously, the seeds were sterilized with 3% sodium hypochlorite for 8 min and washed 5 times with sterile distilled water. Disinfected seeds were placed on Murashige and Skoog basal medium with vitamins 0.5× (PhytoTechnology Laboratories, LLC™, Lenexa, KS, USA) containing 1% agar and 1.5% sucrose (MSA) until germination. The plant experiment was performed in two-compartment Petri dishes (100 × 15 mm) (Greiner Bio-One, Kremsmünster, Austria) containing MSA. Three 4-day-old seedlings were placed on MSA on one side of a two-compartment Petri dish, and the second side had a sterile paper disk of 1 cm diameter (Whatman^®^ filter paper No. 1) impregnated with 10 μL of SLNs containing 2-nonanone or 2-tridecanone at 125, 375, or 500 mg L^−1^. Seedlings without SLNs containing compound exposure were used as a control, and seedlings exposed to only SLN formulation were included as a second control. Petri dishes were sealed with Parafilm^®^, and all assays were performed in 10 replicates of two-compartment Petri dishes. Experimental units were distributed in a completely randomized design and placed in a refrigerated incubator with lighting (VELP Scientifica^®^ FOC 215IL, UsmateVelate, Italy) at 25 °C with a 16:8 day/night photoperiod and 20,000 LUX. The parameters were measured on day 14 [30,33].

### 4.5. Photosynthetic Pigments

The total chlorophyll content on *L. sativa* was performed using 500 mg leaf samples dissolved in 5 mL 80% *v*/*v* aqueous acetone. The absorbance of the extracted chlorophyll was recorded at wavelengths of 470, 649, and 665 nm in the spectrophotometer Genesys 10S UV–VIS (Thermo Scientific^®^, Waltham, MA, USA) [56].

### 4.6. Physiological Parameters

Electrolyte leakage was measured in a solution of 10 mL of deionized water containing 10 leaves, which was incubated at 23 °C for 24 h. Initial electrical conductance (C_1_) was determined using a conductivity meter (PCE Instrument model PHD1,Meschede, Germany). Then, samples were autoclaved, and the final electrical conductivity (C_2_) was determined. The electrolyte leakage (EL) was calculated according to EL (%) = C_1_/C_2_ ×100 [57]. The free proline concentration was determined using 0.5 g of fresh tissue diluted in 70:30 ethanol/water solution. Then, a reaction mix composed of ninhydrin 1% *w*/*v* in acetic acid 60% *v*/*v* and ethanol (20%) was added to samples and a proline calibration curve (0.04–1 mM). Samples and a proline calibration curve were measured at 520 nm in a spectrophotometer, Epoch (Biotek Instrument, Winooski, VT, USA) with the software Gen 5 (version 2.00.18). Total protein concentration was determined according to the Bradford method, where 50 µL of the sample extract (100 mg of leaf tissue in a solution composed of 1 mM ethylenediaminetetraacetic acid (EDTA), 2% *w*/*v* polyvinylpyrrolidone (PVP), and 0.1 mM, pH 7.5 potassium phosphate buffer) was mixed with 750 µL of deionized water and 200 µL of Bradford reactive. Next, samples were incubated at 25 °C for 10 min, and the absorbance was read at 595 nm. The calibration curve was performed with bovine serum albumin (BSA) (10–100 µg) [58].

### 4.7. Determination of Total Phenols

The Folin–Ciocalteu method was used to determine total phenol contents. An amount of 1.5 mL of leaf extract (0.18 g of tissue in 1.5 mL of extraction solvent composed of methanol/water/formic acid, 25:24:3) was added to the mix reaction composed of 1110 µL of deionized water, 75 µL of Folin–Ciocalteu reagent, and 300 µL of 20% *w*/*v* sodium carbonate. Next, samples were incubated at 20 °C under dark conditions for 30 min, and the absorbance was read at 750 nm. Gallic acid at 100 to 500 mg L^−1^ concentrations was used to perform the calibration curve [59].

### 4.8. Identification of Chicoric Acid

Samples were analyzed in high performance liquid chromatography –diode array detection (HPLC–DAD) (Shimadzu, Tokyo, Japan) equipped with an SPD-M20A UV–VIS diode array spectrophotometer (Shimadzu, Duisburg, Germany). Instrument control and data collection were conducted using LabSolutions (Shimadzu, Duisburg, Germany). An Agilent 1100 Series system (Agilent, Waldbronn, Germany) equipped with DAD and an LC/MSD Trap VL (Agilent) electrospray ionization mass spectrometry system coupled to an Agilent ChemStation (version B.01.03) was used to identity assignments [60]. The use of a Kromasil ClassicShell-2.5-C_18_ (4.6 × 100 mm, 2.5 μm) column and a _C18_ precolumn (Novapak; Waters, Milford, MA, USA; 22 × 3.9 mm, 4 μm) was employed for chromatographic separation. The samples were injected using HPLC-grade water, acetonitrile/formic acid (92:3:5 *v*/*v*/*v*) and water/acetonitrile/formic acid (45:50:5 *v*/*v*/*v*) as A and B mobile phases, respectively, with an elution gradient between 6% and 50% B over 30 min at 0.55 mL min^−1^ at 40 °C. Identified assignments were performed in an HPLC-DAD-QTOF-MS/MS compact (Bruker Daltonics GmbH, Bremen, Germany) [61]. Instrument control and data collection were conducted using Compass DataAnalysis 4.4 SR1 (Bruker Daltonics GmbH, Bremen, Germany). Chicoric acid was used as an external standard and quantified at 360 nm (Sigma-Aldrich, Steinheim, Germany).

### 4.9. Antioxidant Capacity

Leaf samples were mashed in liquid nitrogen and dissolved in 1.5 mL of extraction solvent (methanol/water/formic acid, 25:24:3) under darkness. Then, samples were sonicated with an ultrasonic processor at 80% amplitude (Sonics and Materials, Newtown, CT, USA) for 60 s and shaken at 200 rpm for 20 min. After, the samples were centrifuged at 4000× *g* for 20 min, and the supernatant was transferred to another tube and stored at −20 °C [62]. An amount of 240 µL of DPPH radical (0.1 mM) dissolved in methanol was added to a 96-well plate to read the first absorbance. Then, 10 µL of sample or standard was added and incubated under dark conditions for 30 min. Measurements were performed at 517 nm, and the results were expressed as Trolox equivalents. CUPRAC was measured using the following mix: 50 µL of CuCl_2_ (10 mM), 50 µL of neocuproine (7.5 mM), and 50 µL of ammonium acetate buffer at pH 7 (1 M). The mix was added to a 96-well plate and incubated at 27 °C for 30 min under dark conditions. Next, 100 µL of Trolox standard or sample was added and incubated for 30 min at 27 °C. The absorbances were conducted at 450 nm, and the results were expressed as Trolox equivalents. TEAC was performed using aliquots of 245 µL of ABTS 7.5 mM reagent, which was added to a 96-well plate to read the first absorbance at 734 nm. Next, 5 µL of sample or Trolox standard was added to microplate wells and incubated at 30 °C for 30 min to perform the second absorbance reading [59].

### 4.10. Antioxidant Enzyme Activities

Leaves of *L. sativa* seedlings (100 mg) were immersed in liquid nitrogen, and 1 mL of mix solution (1 mM EDTA solution, 2% *w*/*v* PVP, and 0.1 mM, pH 7.5 potassium phosphate buffer) was added to the sample. Next, extracts were centrifuged at 4 °C for 5 min, and the supernatant was used to determine the enzymatic activities, according to Kohatsu et al. [63]. APX activity was determined by adding 50 μL of leave extract to 2.9 mL of aqueous solution (0.1 mM EDTA, 0.5 mM ascorbic acid, 50 mM potassium phosphate buffer at pH 7.0). Then, 30 μL of H_2_O_2_ at 30 mM was added, and the absorbance at 290 nm was read for 3 min. CAT activity was determined by adding 50 μL of leaf extract to 0.95 mL of solution (0.1 mM EDTA, 125 mM H_2_O_2_, and 50 mM of potassium phosphate buffer at pH 7.0). The absorbance was read at 240 nm. POX activity was measured mixing 20 μL of leaf extract with 3.58 aqueous solution (20 mM pyrogallol, 20 mM H_2_O_2_, and 20 Mm potassium phosphate buffer at pH 6.8). The absorbance was measured at 420 nm. SOD activity was determined by mixing 40 μL of leaf extract with 1.96 mL of aqueous solution (0.1 Mm EDTA, 13 Mm methionine, 2 µM riboflavin, 75 µM cloruro de nitroblue tetrazolium (NBT) and 50 mM potassium phosphate buffer at pH 7.8). Next, the samples were exposed to 60 W fluorescent lamp (300 µM m^−2^ s^−1^ of photosynthetically active radiance) for 10 min, and absorbance was read at 560 nm. One unit of SOD was considered to be the amount of the enzyme that inhibited NBT reduction by 50%.

### 4.11. Statistical Analyses

Experimental data were analyzed by Statistix v10 (Tallahassee, FL, USA) to perform analysis of variance (ANOVA). Differences among means of treatments were separated using the Tukey test at 0.05 of significance.

## 5. Conclusions

This study provides clear evidence that SLN is an effective system to implement in agriculture systems for the controlled release of 2-ketones with biological activity. SLNs demonstrated relevant stability over time, maintaining the physicochemical properties and exerting a plant-growth-inducing effect for 14 days. Some doses of 2-nonanone and 2-tridecanone released from SLNs stimulated the growth of *L. sativa* at the root and leaf levels. The results associated with photosynthetic pigments, electrolyte leakage, and proline content suggested that 2-ketones can be a plant stressor agent without altering the growth of *L. sativa*. It notes that the antioxidant capacity enhanced in seedlings exposed to SLN formulation and decreased strongly in treatments with exposure to different doses of 2-ketones, suggesting a specific communication and a possible ecological role between these compounds and plant receptors. Additionally, 2-tridecanone modulates principally the scavenging of H_2_O_2_ by stimulating APX and POX. The results evidenced the capacity of 2-ketones released from SLNs to stimulate the growth of *L. sativa*, modulating physiological processes (i.e., antioxidant system).

## Figures and Tables

**Table 1:** Particle size, polydispersity index (PDI), and ζ-potential of SLNs containing doses of 2-nonanone and 2-tridecanone for 2 months at 25° C. The values are presented as mean ± standard deviation (N = 3). F: SLN formulation.

	2-Nonanone				2-Tridecanone			
	F	125 µL L^−1^	375 µL L^−1^	500 µL L^−1^	F	125 µL L^−1^	375 µL L^−1^	500 µL L^−1^
Month-1								
Size (nm)	352 ± 6.8	306 ±8.4	263 ± 10.7	306 ± 18.6	245 ± 4.6	253 ± 4.0	188± 10.0	261 ± 18.4
PDI	0.39 ± 0.07	0.38 ± 0.03	0.35 ± 0.03	0.35 ± 0.03	0.27 ± 0.01	0.25 ± 0.02	0.26 ± 0.01	0.25 ± 0.03
**ζ**-potential (mV)	−22.4 ± 1.2	−22.9 ± 0.7	−22.1 ± 1.4	−24.3 ± 0.9	−19.2 ± 0.4	−17.9 ± 0.9	−16.0 ± 0.72	−20.0 ± 1.21
Month-2								
Size (nm)	281 ± 4.0	292 ± 30.7	246 ± 10.1	270 ± 11.3	234 ± 4.6	250 ± 4.4	187± 42.4	258 ± 17.6
PDI	0.24 ± 0.01	0.34 ± 0.12	0.28 ± 0.3	0.26 ± 0.2	0.26 ± 0.01	0.25 ± 0.01	0.26 ± 0.04	0.28 ± 0.07
**ζ**-potential (mV)	−20.4 ±0.9	−20.5 ±1.2	−19.2 ±0.4	−21.1 ±0.9	−18.9 ± 0.4	−17.8 ± 1.1	−15.4 ± 2.0	−16.8 ± 3.7

## Data Availability

Not applicable.
